# Consumer adoption of personalised nutrition services from the perspective of a risk–benefit trade-off

**DOI:** 10.1007/s12263-015-0478-y

**Published:** 2015-09-25

**Authors:** Aleksandra Berezowska, Arnout R. H. Fischer, Amber Ronteltap, Ivo A. van der Lans, Hans C. M. van Trijp

**Affiliations:** Department of Social Sciences, Marketing and Consumer Behaviour Group, Wageningen University and Research Centre, Hollandseweg 1, 6706 KN Wageningen, The Netherlands; LEI, Wageningen University and Research Centre, Hollandseweg 1, 6706 KN Wageningen, The Netherlands

**Keywords:** Personalised nutrition, Consumers, Adoption, Privacy Calculus, Service attributes

## Abstract

Through a Privacy Calculus (i.e. risk–benefit trade-off) lens, this study identifies factors that contribute to consumers’ adoption of personalised nutrition services. We argue that consumers’ intention to adopt personalised nutrition services is determined by perceptions of *Privacy Risk*, *Personalisation Benefit*, *Information Control*, *Information Intrusiveness*, *Service Effectiveness*, and the *Benevolence*, *Integrity*, and *Ability* of a service provider. Data were collected in eight European countries using an online survey. Results confirmed a robust and Europe-wide applicable cognitive model, showing that consumers’ intention to adopt personalised nutrition services depends more on *Perceived Personalisation Benefit* than on *Perceived Privacy Risk*. *Perceived Privacy Risk* was mainly determined by perceptions of *Information Control*, whereas *Perceived Personalisation Benefit* primarily depended on *Perceived Service Effectiveness*. Services that required increasingly intimate personal information, and in particular DNA, raised consumers’ *Privacy Risk* perceptions, but failed to increase perceptions of *Personalisation Benefit*. Accordingly, to successfully exploit personalised nutrition, service providers should convey a clear message regarding the benefits and effectiveness of personalised nutrition services. Furthermore, service providers may reduce *Privacy Risk* by increasing consumer perceptions of *Information Control*. To enhance perceptions of both *Information Control* and *Service Effectiveness*, service providers should make sure that consumers perceive them as competent and reliable.

## Introduction

Research within the field of nutrigenomics has raised high expectations, as increased understanding of the genes–nutrition relationship holds the potential to revolutionise disease prevention and health promotion (Arkadianos et al. 2007; Williams et al. 2008). Once it has reached its maturity, nutrigenomics offers the opportunity to prevent disease and promote health through dietary advice tailored to the individual, also referred to as personalised nutrition, rather than homogenous groups within the population (Ghosh 2010). The urge for personalised nutrition is not surprising, as it may not only lead to the most relevant dietary advice, but also stimulate advice adherence (Hurlimann et al. 2014) through increased involvement (Lustria et al. 2009). Consumer reluctance to adopt personalised nutrition may, however, compromise the potential benefits resulting from personalised nutrition.

For consumers, enjoying the benefits of personalised nutrition is practically impossible without getting exposed to some degree of privacy risk, as personalised nutrition advice requires information regarding an individual’s: (1) lifestyle (i.e. questionnaires concerning dietary intake and physical activity), (2) phenotype (i.e. current health status based on, for instance, a blood test), and/or (3) genetic make-up (i.e. DNA profiling based on a buccal swab) (Gibney and Walsh 2013; Rimbach and Minihane 2009). Disclosing these types of personal information to a service provider that generates personalised nutrition advice implies potential negative consequences caused by privacy loss (Mothersbaugh et al. 2012). For instance, consumers may have trouble getting health insurance when their genetic information would be made known to their insurance company. Hence, consumers’ willingness to disclose personal information in return for the benefits of personalised nutrition advice, while putting at risk their privacy, is considered decisive in the adoption of personalised nutrition.

Although highly relevant for the domain of nutrition and health, consumers’ intention to engage in personalisation is most often studied in business-related contexts such as advertising and e-commerce (e.g. Li and Unger 2012; Taylor et al. 2009; van Doorn and Hoekstra 2013). Due to a difference in the intimacy level of the required personal information (e.g. demographics and purchase history vs. health information), it cannot be assumed that the findings from the business context are fully applicable to personalised nutrition. Hence, to successfully exploit personalised nutrition, knowledge on factors that contribute to consumers’ adoption of personalised nutrition is required. The current study, therefore, aims to provide insight into determinants of consumers’ intention to adopt personalised nutrition.

### Theoretical framework

**Fig. 1:**
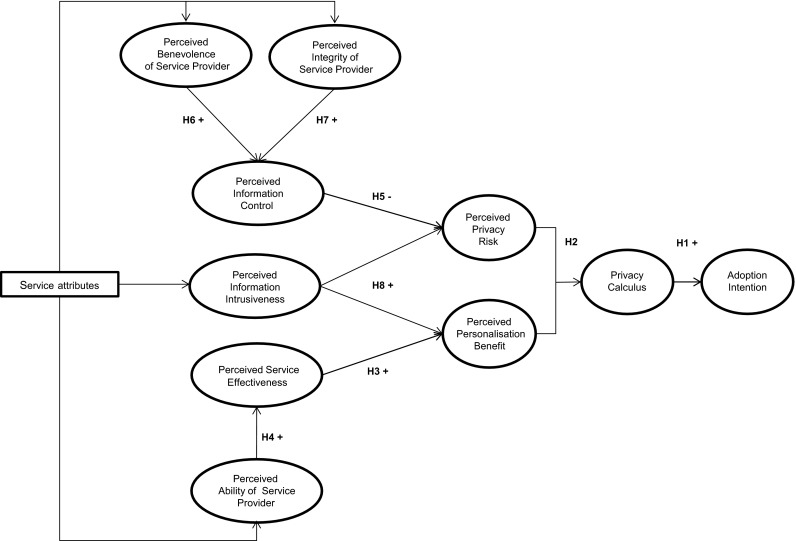
Theoretical framework

The theoretical framework (Fig. 1) of this study proposes consumers’ intention to adopt personalised nutrition to be determined by the shared impact of risk and benefit perceptions (Berezowska et al. 2014). The balance between desired benefits and undesired risks is assessed by combining risk and benefit perceptions into an overall information disclosure valuation (Li 2012), captured by the *Privacy Calculus* (Culnan and Armstrong 1999). The *Privacy Calculus* builds on the principles of behavioural decision-making theories (e.g. Blau 1964; Kahneman and Tversky 1979; Vroom 1964) in assuming that consumers behave in ways that maximise positive outcomes (i.e. benefits) and minimise negative outcomes (i.e. risks) resulting from information disclosure (Keith et al. 2013). Hence, consumers will only be willing to adopt personalised nutrition, rather than general dietary advice, if the perceived benefits of information disclosure offset the perceived risks of information disclosure (Dinev and Hart 2006). When the outcome of the *Privacy Calculus* is positive (i.e. perceived benefits are greater than perceived risks), consumers are more inclined to disclose personal information for the purpose of personalisation. In contrast, a negative *Privacy Calculus* outcome (i.e. perceived benefits are lower than perceived risks) is likely to result in the rejection of personalised nutrition (Xu et al. 2011). Therefore, we hypothesise that:

#### *Hypothesis* 1

The more positive the outcome of the *Privacy Calculus*, the more likely consumers are to adopt personalised nutrition services.

As risks and benefits of information disclosure for the purpose of personalisation generally revolve around privacy risks and personalisation benefits, we presume that the key drivers of the *Privacy Calculus* outcome will be consumer perceptions of *Personalisation Benefit* and *Privacy Risk*:

#### *Hypothesis* 2

The *Privacy Calculus* outcome is determined by perceptions of both *Privacy Risk* and *Personalisation Benefit*.

*Personalisation Benefit* can be viewed in terms of the personal value that consumers perceive to receive in return for information disclosure (Chellappa and Sin 2005). The value of personalised nutrition is, amongst others, based on the extent to which an individual expects that using personalised nutrition will help him/her to attain a particular goal (e.g. improve health) (Sweeney and Soutar 2001). Consumer perceptions of value, therefore, depend on the effectiveness of personalised nutrition, which is rooted in concepts such as usefulness (Davis 1989) and expected performance (Venkatesh et al. 2003). The extent to which consumers perceive engaging in personalised nutrition as effective is affected by a service provider’s *Ability* to transform the acquired personal information into a tailored and useful advice. That is to say, believing that a service provider is able to transform personal information into effective personalised nutrition advice assures consumers that engaging in personalised nutrition will enable them to achieve their goal (Earle 2010; Siegrist et al. 2005). Therefore, service providers who prompt higher levels of *Perceived Ability* will be seen as suppliers of more effective services, which in turn will increase consumers’ perception of *Personalisation Benefit*. Thus, we suggest that:

#### *Hypothesis* 3

*PerceivedPersonalisation Benefit* increases with increasing perceptions of *Service Effectiveness*.

#### *Hypothesis* 4

*PerceivedService Effectiveness* increases with increasing perceptions of a service provider’s *Ability*.

*Privacy Risk* perceptions are determined by the extent to which consumers believe that privacy loss is likely to occur (Smith et al. 2011). Perceptions of likely privacy loss are reduced if consumers feel in control of which personal information is disclosed and how the disclosed information is being used (Phelps et al. 2000). Hence, *Information Control* mitigates *Perceived Privacy Risk* by making consumers feel in control of the privacy risk they are exposed to (Margulis 2003). Consumer perceptions of *Information Control* result from the belief that a service provider is trustworthy and consequently will not misuse the disclosed personal information. If consumers perceive a service provider to be a person of *Benevolence* (i.e. wants to do good) and *Integrity* (i.e. adheres to sound moral and ethical principles), high perceptions of trustworthiness are in place (Colquitt et al. 2007). Therefore, service providers who induce high perceptions of *Benevolence* and *Integrity* are likely to increase consumer perceptions of *Information Control* and with that reduce consumer perceptions of *Privacy Risk*:

#### *Hypothesis* 5

*PerceivedPrivacy Risk* decreases with increasing perceptions of *Information Control*.

#### *Hypothesis* 6

*PerceivedInformation Control* increases with increasing perceptions of a service provider’s *Benevolence*.

#### *Hypothesis* 7

*PerceivedInformation Control* increases with increasing perceptions of a service provider’s *Integrity*.

Both *Privacy Risk* and *Personalisation Benefit* perceptions are likely to depend on the personal information that is required for personalisation to take place. Personal information allowing for personalisation varies in breadth and depth (Taddei and Contena 2013). Information breadth denotes the quantity of the required information, whereas information depth refers to the intimacy level of the information (Lee et al. 2013). Based on the extent to which the information is perceived to approach an individual’s core identity, personal information can be classified into four categories (Marx 2005) that increase in intimacy level: (1) individual information (e.g. demographics), (2) private information (e.g. lifestyle), (3) sensitive information (e.g. health status), and (4) unique information (e.g. DNA). The more information is required and the higher the intimacy level of this information, the greater the intrusiveness of the personal information. Consumers’ concern regarding information disclosure increases as personal information becomes more intrusive (Goldsmith et al. 2012; Li et al. 2011; Sheehan and Hoy 2000). At the same time, an increase in *Information Intrusiveness* leads to more effective personalised nutrition advice. Hence, the more intrusive the required personal information, the more likely it becomes that personalisation will result in valuable benefits, but also the more severe the consequences of possible privacy loss (Wendel et al. 2013). Consequently, we hypothesise that:

#### *Hypothesis* 8

Both *Perceived Personalisation Benefit* and *Perceived Privacy Risk* increase with increasing perceptions of *Information Intrusiveness*.

Once the cognitive process behind consumers’ intention to adopt personalised nutrition has been mapped, it is important to identify factors that drive this cognitive process. Looking at personalised nutrition as an information exchange process (van Trijp and Ronteltap 2007), it becomes clear that the cognitive process behind consumers’ intention to adopt personalised nutrition is fuelled by attributes that shape the way in which personalised nutrition advice is generated and provided. The information exchange process consists of three consecutive stages: (1) the consumer discloses personal information to a service provider; (2) the service provider uses the personal information to generate personalised nutrition advice; and (3) the service provider provides the personalised nutrition advice to the consumer (Ronteltap et al. 2013). Although personal information remains at the heart of personalised nutrition, the information exchange process suggests that service attributes such as communication mode, service scope, and service frequency also contribute to consumers’ intention to adopt personalised nutrition. Consumers may, for instance, be reluctant to disclose DNA to a service provider that limits himself to email communication (Metzger 2004) or perceive information disclosure as more valuable when nutrition advice is provided more than once (Seiders et al. 2014). Since consumers’ preference for and reaction to service attributes may differ from country to country (Pullman et al. 2001), to consolidate widespread adoption of personalised nutrition, it is important to identify which service attributes amplify or mitigate adoption intention across different countries.

## Methods

### Sample and procedure

To test the theoretical model, a total of 8136 participants from eight European countries (Greece, Spain, the Netherlands, Ireland, the UK, Germany, Poland, and Norway) participated in the study. To ensure nationally representative samples, participants were quota-sampled based on gender, age, region of residence, and highest level of education completed according to the International Standard Classification of Education (UNESCO Institute for Statistics 2012). Participants’ age was 41 years on average and ranged from 18 to 65. The sample included 49.9 % men. Of all participants, 29.9 % enjoyed tertiary education, 40.5 % obtained an upper-secondary or post-secondary education degree, and 30.5 % completed lower-secondary education or less.

Participants were sampled from the panels of a market research agency (GfK) and invited to participate in the survey by email. Completion of the online survey took about 18 min. The overall response rate was 51 %. To compensate for time and effort, participants were rewarded credits that accumulate to a gift voucher. Data were collected in November/December 2013.

### Stimuli

**Fig. 2:**
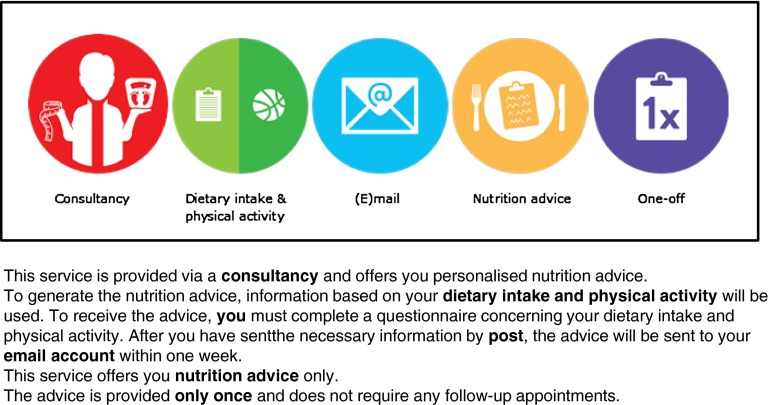
Representation of personalised nutrition service descriptions

Fictitious personalised nutrition services were used as stimulus material. A total of 144 services were generated using a full-factorial design combining five service attributes (personal information with four levels, service provider with three levels, communication mode with two levels, advice scope with three levels, and advice frequency with two levels) based on Berezowska et al. (2014) (Table 1). Each participant was shown two personalised nutrition services. To ensure intra-individual variance in the *Information Intrusiveness* construct, the two personalised nutrition services contained different levels of personal information. Taking account of this condition, the first personalised nutrition service was assigned completely at random, while the second personalised nutrition service was assigned partially at random. For instance, if the first service required DNA through the collection of a buccal swab, the second service had to require lifestyle information, phenotypic information through the collection of a blood sample, or the combination of phenotypic information and DNA. The service attribute levels of both personalised nutrition services were presented to the participants using pictograms supported by textual descriptions (Fig. 2). To control for assumptions regarding terms and conditions, participants were told that all services met the guidelines of the European Association of Dietitians (a non-existent organisation). Furthermore, to ensure that all services were evaluated from the same perspective, participants were instructed to imagine being in need of a service that could help them develop a healthier lifestyle.

**Table 1:** Personalised nutrition service attributes and levels

Service attribute	Service attribute levels
Personal information	Low-quantity private information: Lifestyle Mid-quantity sensitive information: Lifestyle + Phenotype Mid-quantity unique information: Lifestyle + DNA High-quantity unique information: Lifestyle + Phenotype + DNA
Service provider	Consultancy + dietician Fitness club + dietician Employer + dietician
Communication mode	No personal contact Personal contact
Advice scope	Nutrition advice Nutrition advice + Exercise advice Nutrition advice + Exercise advice + Group support meetings
Advice frequency	One-off Monthly

^a^All services required contact details (name, address) and individual information (height, weight, gender, and age)

^b^Lifestyle = dietary intake and physical activity

### Measures

Measures were derived from existing scales adapted from prior studies (Table 2). As no relevant *Information Intrusiveness* scale was available, *Information Intrusiveness* items were developed based on Zwick and Dholakia (2004). All items were answered on seven-point scales ranging from strongly disagree to strongly agree or, in case of the *Privacy Calculus*, greater risks to greater benefits. The survey was pretested in the Netherlands using cognitive walkthrough interviews (*N* = 12). Based on the pretest minor amendments related to the questionnaire’s layout and comprehensiveness of the personalised nutrition service descriptions were made. To test the adequacy of the revised questionnaire, an online pilot study was conducted in the UK (*N* = 50) and the Netherlands (*N* = 50). The pilot study did not result in further amendments. Finally, the English questionnaire was translated and back-translated (Brislin 1970) into the national languages of the participating countries.

**Table 2:** Measures

Construct	Adapted from	Question	Items	Anchors
Adoption Intention	Zarmpou et al. (2012)/Kim and Park (2013)		I would consider using this service I intend to use this service I would recommend this service to others	1 = “Strongly disagree” to 7 = “Strongly agree”
Privacy Calculus	Xu et al. (2011)		All things considered, do you think using Service 1^a^ will offer greater benefits than risks, or greater risks than benefits	1 = “Greater risks” to 7 = “Greater benefits”
Personalisation Benefit	Xu et al. (2009)	Compared to general nutrition advice, Service 1 offers me nutrition advice that is	More accurately tailored to my health needs More relevant for my health More beneficial for my health	1 = “Strongly disagree” to 7 = “Strongly agree”
Privacy Risk	Xu et al. (2009)	I think that using Service 1	Involves many privacy-related risks Is a threat to my privacy Creates a high risk for the loss of my privacy	1 = “Strongly disagree” to 7 = “Strongly agree”
Information Intrusiveness	Developed based on Zwick and Dholakia (2004)	The way in which Service 1 obtains my personal information results in	Correct information Accurate information Detailed information	1 = “Strongly disagree” to 7 = “Strongly agree”
Service Effectiveness	Davis (1989)/Venkatesh et al. (2003)	Service 1	Enables me to develop a healthier lifestyle Helps me to have a healthier lifestyle Makes me feel in control of developing a healthier lifestyle	1 = “Strongly disagree” to 7 = “Strongly agree”
Information Control	Mothersbaugh et al. (2012)	The way in which Service 1 will use my personal information	Is completely determined by me Depends completely on me giving my approval Is under my control	1 = “Strongly disagree” to 7 = “Strongly agree”
Ability of Service Provider	Mayer and Davis (1999)	I think that the provider of Service 1	Is very capable of providing personalised nutrition advice Has much knowledge about personalised nutrition advice Has the skills to provide personalised nutrition advice	1 = “Strongly disagree” to 7 = “Strongly agree”
Benevolence of Service Provider	Mayer and Davis (1999)	I think that the provider of Service 1	Is very concerned about my welfare Will not knowingly do anything to hurt me Looks out for what is important to me Will go out of its way to help me	1 = “Strongly disagree” to 7 = “Strongly agree”
Integrity of Service Provider	Mayer and Davis (1999)	I think that the provider of Service 1	Sticks to its word Tries to be fair in dealing with others Is guided by sound principles	1 = “Strongly disagree” to 7 = “Strongly agree”

^a^“Service 1” was replaced with “Service 2” when evaluating the second personalised nutrition service description

### Data analysis

The proposed model was tested using confirmatory factor analysis and structural equation modelling with maximum likelihood estimation in the R package lavaan (Rosseel 2012).

First, to rule out the possibility of language causing differences between countries, the relationship between a latent construct and its items (i.e. measurement model) was assessed through a multi-group confirmatory factor analysis. Using one-factor models, cross-national equivalence of the employed measures was established on the basis of three consecutive tests (Steenkamp and Baumgartner 1998) for each latent construct individually. Test 1 checked whether the items of a particular measure loaded on the same latent construct in all countries, meaning that the conceptual definition of a latent construct was similar across countries (i.e. configural invariance). Test 1 was conducted for *Perceived Benevolence of Service Provider* only, given that, in the light of model identification, assessing configural invariance for one-factor models is solely meaningful when construct scales consist of at least four items (Brown 2006). Test 2 assessed whether the factor loadings of a particular item were equal across countries, indicating that a latent construct has the same meaning in all countries (i.e. metric invariance). Test 3 established whether the average item scores were equivalent across countries, showing that response patterns were equal across countries (i.e. scalar invariance). When cross-national equivalence was not reached, parameters related to configural, metric, and/or scalar invariance were relaxed based on the modification indices.

Second, to determine whether scalar invariance could be assigned to the overall measurement model, Test 3 was repeated using a multi-factor model consisting of all latent constructs and their items, while accounting for the relaxations suggested by the one-factor models.

Third, internal consistency of the latent constructs was evaluated on the basis of two reliability checks: (1) ω^2^, adequate when >0.7 (Nunnally 1978); and (2) average variance extracted (AVE), adequate when >0.5 (Fornell and Larcker 1981). Discriminant validity (i.e. the extent to which the measured constructs are distinct) was confirmed when the shared variation between a construct and its items (i.e. AVE) exceeded the shared variance between that particular construct and each of the other constructs (Fornell and Larcker 1981).

Fourth, the causal relations between the latent constructs (i.e. structural model) were assessed. To identify differences and similarities between countries, a multi-group structural equation model was performed. The structural model was tested in six steps that consecutively added equality constraints across countries: Step 1) strength of causal relation (i.e. path coefficient or β) between latent constructs is allowed to vary across countries; Step 2) strength of causal relation between latent constructs is not allowed to vary across countries; Step 3) variances and covariances amongst exogenous latent constructs *Ability*, *Benevolence*, *Integrity*, and *Information Intrusiveness* are not allowed to vary across countries; Step 4) regression intercepts for *Information Control*, *Service Effectiveness*, *Privacy Risk*, *Personalisation Benefit*, *Privacy Calculus*, and *Adoption Intention* are not allowed to vary across countries; Step 5) means for *Ability*, *Benevolence*, *Integrity*, and *Information Intrusiveness* are not allowed to vary across countries; and Step 6) the extent to which an explanatory variable explains an outcome variable is not allowed to vary across countries (i.e. *R*^2^).

To determine whether both the measurement model and structural model were equal across countries, model fit was assessed based on four goodness of fit indices: (1) root mean square error of approximation (RMSEA), good if <.07; (2) standardised root mean square residual (SRMR), good if <0.08; (3) Comparative Fit Index (CFI), good if >0.95; and (4) Tucker-Lewis index (TLI), good if >0.95. The adopted cut-off values were derived from Hair et al. (2010).

To evaluate the main effects of the service attributes corrected for population variance, the individual cases (*N* = 16,272) were aggregated into 144 new cases representing each of the 144 personalised nutrition services. The aggregated data were analysed using multivariate analysis of variance with the service attributes as explanatory variables and *Privacy Risk*, *Personalisation Benefit*, *Privacy Calculus*, and *Adoption Intention* as outcome variables.

## Results

### Measurement model

To rule out the possibility of language causing differences between countries, the relationships between the different latent construct and their items were subjected to several tests.

Partial configural invariance was confirmed for *Perceived Benevolence of Service Provider,* implying that its conceptual definition was similar across countries (Table 3). Partial configural invariance for *Perceived Benevolence of Service Provider* was reached by introducing error covariance between item 1 (concerned about welfare) and item 4 (goes out of its way to help).

**Table 3:** Fit measures for the one-factor multi-item models and the overall measurement model

	Scalar invariance	Chi-square	*df*	CFI	TLI	RMSEA			SRMR
						Value	90 % LB	90 % UB	
*One*-*Factor Models*									
Adoption Intention	Partial^a^	344.92	27	0.992	0.992	0.076	0.069	0.083	0.030
Personalisation Benefit	Yes	90.50	28	0.999	0.999	0.330	0.026	0.041	0.013
Privacy Risk	Yes	208.01	28	0.997	0.99	0.056	0.048	0.063	0.018
Information Intrusiveness	Yes	219.54	28	0.996	0.996	0.058	0.051	0.065	0.027
Service Effectiveness	Yes	79.57	28	0.999	0.999	0.030	0.022	0.028	0.010
Information Control	Yes	275.22	28	0.994	0.995	0.066	0.059	0.073	0.034
Ability of Service Provider	Yes	107.63	28	0.999	0.999	0.037	0.030	0.045	0.011
Benevolence of Service Provider	Partial^b^	692.80	51	0.988	0.988	0.079	0.074	0.084	0.048
Integrity of Service Provider	Yes	211.13	28	0.996	0.997	0.057	0.050	0.064	0.019
Overall Measurement Model	Partial^c^	14,264.38	2922	0.980	0.977	0.044	0.043	0.044	0.032

^a^Equality of item intercept relaxed for item 1 in Poland

^b^Model includes error covariance between item 1 and item 4, which is equal across countries except Norway. Equality of item intercept relaxed for item 1 in Spain, Poland, and The Netherlands. Equality of item intercept relaxed for item 2 in Norway and Poland

^c^Including error covariance and intercept relaxations identified in the one-factor measurement models

Metric invariance was achieved for all multi-item constructs, except *Perceived Benevolence of Service Provider,* indicating that the latent constructs have the same meaning in all countries. Partial metric invariance for *Perceived Benevolence of Service Provider* was reached after relaxing the equality constrain for the error covariance between item 1 and item 4 in the case of Norway.

Demonstrating equal response patterns across countries, scalar invariance was achieved for *Perceived Integrity of Service Provider*, *Perceived Ability of Service Provider*, *Perceived Information Control*, *Perceived Information Intrusiveness, Perceived Service Effectiveness*, *Perceived Privacy Risk*, and *Perceived Personalisation Benefit*. After relaxing some equality constraints (see Table 3), partial scalar invariance was obtained for *Perceived Benevolence of Service Provider* and *Adoption Intention.* After relaxing the relevant parameters, CFI, TLI, and SRMR showed good fit for all constructs. The RMSEA indicated good fit for all constructs except *Perceived Benevolence of Service Provider* (RMSEA = 0.079) and *Adoption Intention* (RMSEA = 0.076). These RMSEA values could, however, be considered sufficiently close to good fit at this stage (Baumgartner and Homburg 1996).

Given that the *Privacy Calculus* was a single-item construct, establishing configural, metric, and scalar invariance was irrelevant. Furthermore, measuring the *Privacy Calculus* with only one item made estimating the item’s error variance impossible. To distribute variance between the latent construct and the item, the error variance of the single-item construct *Privacy Calculus* was set to 20 % (Fuchs and Diamantopoulos 2009).

Since the CFI, TLI, RMSEA, and SRMR values for the overall measurement model indicated good fit (Table 3), it can be assumed that despite the difference in language, the measurement model is equal across all participating countries.

All constructs fulfilled the requirements for internal consistency. The ω^2^ values ranged from 0.888 to 0.969. The AVE values ranged from 0.712 to 0.913. Discriminant validity was adequate across all constructs except *Benevolence of Service Provider*. *Benevolence of Service Provider* was not distinct from *Integrity of Service Provider* in the case of Norway, Germany, Greece, Poland, and the Netherlands. Nevertheless, considering the (1) evidence for discriminant validity of the two constructs in the other countries, (2) AVE for *Integrity of Service Provider* (0.816–0.876) being considerably larger than the between-construct variance (0.757–0.799), and (3) almost identical values of the AVE for *Benevolence of Service Provider* (0.712–0.772) and the between-construct variance (0.757–0.799), it was decided that *Benevolence of Service Provider* and *Integrity of Service Provider* would not be merged.

### Structural model

Table 4 shows the fit measures for the six consecutive steps based on which differences and similarities between the causal relations across countries were assessed. Although most fit measures met the proposed cut-off values, the SRMR values were slightly higher than the recommended cut-off criterion. As adding relations would diminish the parsimony of our model and introduce empirically determined rather than theoretical relations, it was decided to not adjust the model.

**Table 4:** Fit measures for the six steps of the structural equation model

Step	Chi-square	*df*	CFI	TLI	RMSEA			SRMR
					Value	90 % LB	90 % UB	
1. Varying path coefficients^a^	26,957.51	4954	0.960	0.957	0.047	0.046	0.047	0.089
2. Equal path coefficients	27,746.81	5276	0.959	0.959	0.046	0.045	0.046	0.093
3. Equal (co-) variances amongst Ability, Benevolence, Integrity, Information Intrusiveness	28,454.41	5346	0.958	0.958	0.046	0.046	0.047	0.102
4. Equal regression intercepts	29,523.92	5381	0.956	0.957	0.047	0.046	0.047	0.099
5. Equal means Ability, Benevolence, Integrity, Information Intrusiveness	29,960.09	5409	0.956	0.955	0.047	0.047	0.048	0.101
6. Equal *R* ^2^	30,879.62	5451	0.954	0.955	0.048	0.047	0.048	0.102

^a^Step 1 included covariances between Ability, Benevolence, Integrity, and Information Intrusiveness

Correlations between *Ability of Service Provider, Benevolence of Service Provider, Integrity of Service Provider*, and *Information Intrusiveness* were high and ranged from 0.64 to 0.87 (*p* < 0.001).

### Hypothesis testing

**Fig. 3:**
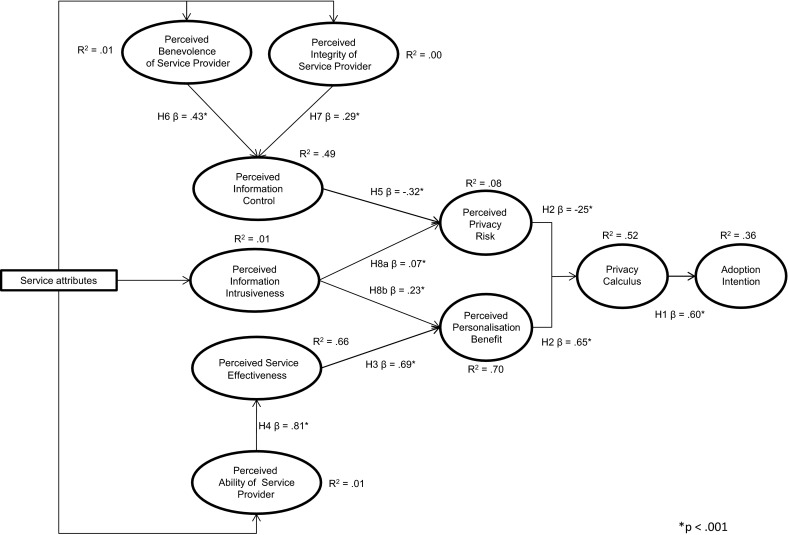
Final structural model

The first important finding is the fact that all hypothesised relations were significant and equal across countries (Fig. 3). In addition, the extent to which the model explained *Information Control*, *Service Effectiveness*, *Personalisation Benefit*, *Privacy Calculus*, and *Adoption Intention* was substantial, as the proportions of explained variance ranged from 36 to 70 %. With 8 %, the explained variance of *PerceivedPrivacy Risk* was modest (*R*^2^ = 0.08).

As expected based on Hypothesis 1, *Adoption Intention* was determined by the outcome of the *Privacy Calculus*. The more positive the outcome of the *Privacy Calculus*, the higher the participants’ intention to adopt personalised nutrition services (*β* = .60; *p* < .001). Confirming Hypothesis 2, the outcome of the *Privacy Calculus* depended on both *Privacy Risk* and *Personalisation Benefit* perceptions. *PerceivedPrivacy Risk* had a negative effect on the outcome of the *Privacy Calculus* (*β* = −.25; *p* < .001), while *PerceivedPersonalisation Benefit* had a positive effect on the outcome of the *Privacy Calculus* (*β* = .65; *p* < .001). Compared to the path coefficient of *Privacy Risk*, the path coefficient of *Personalisation Benefit* was almost three times as high.

Confirming Hypothesis 3 and Hypothesis 8b, *PerceivedPersonalisation Benefit* depended on participants’ perceptions of *Service Effectiveness* and *Information Intrusiveness.PerceivedService Effectiveness* and *PerceivedInformation Intrusiveness* were positively related to *Perceived Personalisation Benefit,* meaning that an increase in both *Service Effectiveness* (*β* = .69; *p* < .001) and *Information Intrusiveness* (*β* = .23; *p* < .001) results in higher perceptions of *Personalisation Benefit*. Comparing the path coefficients of *PerceivedService Effectiveness* and *Perceived Information Intrusiveness*, the effect of *PerceivedService Effectiveness* on *PerceivedPersonalisation Benefit* was three times as high. In line with Hypothesis 4, *PerceivedService Effectiveness* depended on the *Perceived Ability of the Service Provider.* As the *PerceivedAbility of the Service Provider* rose, so did participants’ perceptions of *Service Effectiveness* (*β* = .81; *p* < .001).

In line with Hypothesis 5 and Hypothesis 8a, *PerceivedPrivacy Risk* was affected by both *Perceived Information Intrusiveness* and *PerceivedInformation Control*. The relation between *Information Intrusiveness* and *PerceivedPrivacy Risk* was positive (*β* = .07; *p* < .001), indicating that an increase in *Information Intrusiveness* caused an increase in the perception of *Privacy Risk*. The influence of *PerceivedInformation Intrusiveness* on *PerceivedPrivacy Risk* was, however, minor. In the case of *PerceivedInformation Control*, participants’ perception of *Privacy Risk* decreased as perception of *Information Control* increased (*β* = −.32; *p* < .001). Consistent with Hypothesis 6 and Hypothesis 7, *Perceived Information Control* was determined by both *PerceivedBenevolence of the Service Provider* and *PerceivedIntegrity of the Service Provider*. An increase in both *Benevolence* (*β* = .43; *p* < .001) and *Integrity* (*β* = .29; *p* < .001) enhanced participants’ perceptions of *Information Control.*

The impact of the service attributes on the cognitive process behind consumers’ intention to adopt personalised nutrition was minor. Although most of the service attributes had a significant effect on the *PerceivedAbility of the Service Provider*, *Benevolence of the Service Provider*, *Integrity of the Service Provider*, and *Information Intrusiveness,* the extent to which the service attributes explained each of these latent constructs was approximately 1 % (Table 5). Aggregated data showed that *Adoption Intention* was affected by *Personal Information*, *Service Provider,* and *Communication Mode.* The outcome of the *Privacy Calculus* was influenced by all service attributes except *Advice Scope*. Perceptions of *Privacy Risk* were induced by *Personal Information* and the *Service Provider*. Disclosing unique information (i.e. DNA) and services offered by an employer was perceived as most risky, whereas private information (i.e. lifestyle) and services offered by a fitness clubs was perceived as least risky. *PerceivedPersonalisation Benefit* resulted from the service attributes *Advice Scope*, *AdviceFrequency*, and *Service Provider*. Nutrition and exercise advice that was offered on a monthly basis by a fitness club was perceived as most beneficial. Communicating by means of personal contact had a positive effect on the *Privacy Calculus* and *Adoption Intention* as it reduced *Privacy Risk* perceptions and increased *Personalisation Benefit* perceptions (Table 6).

**Table 5:** Path coefficients of service attribute levels

Service attribute	Construct			
	Ability of service provider	Benevolence of service provider	Integrity of service provider	Information Intrusiveness
*Personal information*				
Phenotype (compared to lifestyle)	0.016	0.003	0.006	0.044*
DNA (compared to lifestyle)	−0.035	−0.064**	−0.085***	0.045*
Phenotype × DNA (compared to lifestyle)	0.006	−0.049*	−0.056*	0.080***
*Service provider*				
Fitness club (compared to consultancy)	−0.005	0.068**	0.047*	−0.005
Employer (compared to consultancy)	−0.031	−0.052*	−0.011	−0.012
*Communication mode*				
Personal contact (compared to no personal contact)	0.130***	0.109***	0.089***	0.114***
*Advice scope*				
Nutrition + exercise (compared to nutrition only)	0.021	0.053**	0.022	0.015
Nutrition + exercise + support group (compared to nutrition only)	−0.002	0.024	0.011	0.012
*Advice frequency*				
Monthly (compared to one-off)	0.058***	0.050**	0.029	0.047**

* *p* < 0.05; ** *p* < 0.01; *** *p* < 0.001

**Table 6:** Estimated marginal means of the service attribute levels for Privacy Risk, Personalisation Benefit, Privacy Calculus, and Adoption Intention

Service attribute	Construct			
	Privacy risk	Personalisation Benefit	Privacy Calculus	Adoption Intention
*Personal information*				
Lifestyle	3.86^a^	4.70	4.74^b^	4.19^c^
Phenotype	3.97^b^	4.71	4.73^b^	4.17^bc^
DNA	4.16^c^	4.65	4.61^a^	4.01^a^
Phenotype × DNA	4.15^c^	4.69	4.60^a^	4.09^ab^
*Service provider*				
Consultancy	3.98^a^	4.68^ab^	4.67^b^	4.05^a^
Fitness club	3.91^b^	4.73^b^	4.79^c^	4.19^b^
Employer	4.22^c^	4.65^a^	4.55^a^	4.10^a^
*Communication mode*				
No personal contact	4.12^a^	4.60^a^	4.57^a^	4.06^a^
Personal contact	3.95^b^	4.77^b^	4.77^b^	4.17^b^
*Advice scope*				
Nutrition	4.04	4.66^a^	4.65	4.10
Nutrition + exercise	4.01	4.73^b^	4.70	4.15
Nutrition + exercise + support group	4.06	4.67^a^	4.66	4.09
*Advice frequency*				
One-off	4.01	4.65^a^	4.63^a^	4.11
Monthly	4.06	4.73^b^	4.71^b^	4.12

Within a particular construct, means sharing the same superscript are not significantly different from the other levels of the same service attribute at *p* < .05 Tukey HSD

## Discussion

This study developed and tested a comprehensive model explaining consumers’ intention to adopt personalised nutrition services. Confirming all hypothesised relations, we find strong support for the proposed model. Moreover, we show that the basic model structure is generalisable to eight European countries. Together, these findings point towards a robust and Europe-wide applicable cognitive model that predicts differences in consumers’ intention to adopt personalised nutrition.

The proposed cognitive model postulates a central role for the *Privacy Calculus* in consumers’ intention to adopt personalised nutrition services. Most studies that assume the *Privacy Calculus* to mediate the relationship between risk and benefit perceptions on the one hand and intention on the other, do not explicitly measure the outcome of such calculus (e.g. Dinev et al. 2013; Keith et al. 2013; Xu et al. 2013). Reasons for omitting an explicit *Privacy Calculus* measure may stem from the belief that the *Privacy Calculus* does not contribute beyond perceptions of *Privacy Risk* and *Personalisation Benefit*. The current study, however, suggests that including an explicit *Privacy Calculus* measure supports the understanding of *Adoption Intention* without affecting the explanatory power of risk and benefit perceptions. Including an explicit *Privacy Calculus* measure in addition to *Privacy Risk* and *Personalisation Benefit* measures is, therefore, recommended.

The *Privacy Calculus* depends more on consumer perceptions of *Personalisation Benefit* than on perceptions of *Privacy Risk*. The dominant role of *Perceived Personalisation Benefit* is in line with the “privacy paradox” (e.g. Bélanger and Crossler 2011; Pavlou 2011; Smith et al. 2011), which implies that consumers tend to put their privacy concerns aside if they expect information disclosure to result in attractive benefits. As most consumers perceive products and services that are tailored to their specific needs to be beneficial (e.g. Franke et al. 2009; Kalyanaraman and Sundar 2006), it is likely that the effect of *Privacy Risk* perceptions on the *Privacy Calculus* may have been offset by perceptions of *Personalisation Benefit*.

Our findings show that disclosing increasingly intimate personal information did not result in higher perception of *Personalisation Benefit*, but did increase perceptions of *Privacy Risk.* This suggests that consumers are aware of the *Privacy Risk* that is induced by the disclosure of highly intimate personal information (i.e. DNA), but not of the *Personalisation Benefit.* In the light of the *Privacy Calculus,* this would mean that the benefits resulting from disclosing highly intimate personal information may not suffice to offset the risk associated with the disclosure of highly intimate information. Such risk–benefit balance is likely to lead consumers towards “intermediate” levels of personalised nutrition that are less intrusive but also less effective. Hence, although studies into DNA-based personalised nutrition advice report consumers to favour personalised over general nutrition advice (e.g. Nielsen and El-Sohemy 2012; Nielsen et al. 2014), we should not lose sight of the role that *Privacy Risk* plays in consumers’ intention to adopt personalised nutrition. To offset *Privacy Risk* perceptions, service providers may even need to educate consumers about the benefits of DNA-based personalised nutrition, over and above those of lifestyle- and phenotype-based personalised nutrition.

Compared to the other latent constructs included in our theoretical model, the explained variance of *PerceivedPrivacy Risk* was modest. Reasons for this low percentage of explained variance in the *Privacy Risk* construct may be twofold. First, the applied methodology may have induced a non-committal way of consumers expressing their *Adoption Intention*, which may have inhibited participants from taking a closer look at *Privacy Risk* determinants such as *Information Control* and *Information Intrusiveness*. Hence, in situations where the decision to engage with a personalised nutrition service is no longer hypothetical (Hofstetter et al. 2013), the effect of *PerceivedInformation Control* and *Perceived Information Intrusiveness* on *Privacy Risk* perceptions may be larger than would be expected on the basis of the current findings (Trope and Liberman 2010). Second, the specific operationalisation of privacy risk may have steered respondents towards privacy risk determinants related to information exchange, rather than those related to information management (Hong and Thong 2013). Information management-related privacy concerns such as unauthorised access due to inadequate information storage security (Anton et al. 2010) may provide additional insight into consumers’ *Privacy Risk* perception (Cortese and Lustria 2012; Smith et al. 1996; Zhou 2011). Future research is recommended to include both information exchange and information management-related determinants of *Privacy Risk*.

With regard to the trust dimensions (Mayer and Davis 1999), *Perceived Ability of the Service Provider* (i.e. competence) had a large effect on *PerceivedService Effectiveness* and through that on consumer perceptions of *Personalisation Benefit*. Furthermore, *Perceived Benevolence* and *Integrity of the Service Provider* (i.e. reliability) influenced *PerceivedInformation Control* and through that *PerceivedPrivacy Risk*. In the current analysis, we followed the idea that each of the trust dimensions has a distinct contribution to the decision process (Colquitt et al. 2007; Terwel et al. 2009). That is, competence-related trust dimensions may be associated with consumers’ confidence in service effectiveness (Earle 2010; Siegrist et al. 2005), while reliability-related trust dimensions may be linked to social trust that comprises the belief whether service providers can be relied on when it comes to having control over personal information (Earle and Cvetkovich 1995). Although the current findings support the idea of the different trust dimensions playing a distinct role in the decision-making process, we cannot be conclusive about how the different trust dimensions are best positioned in the hypothesised model. Future research should, therefore, systematically test the relevance of each trust dimension on the different latent constructs.

Considering the extent to which the proposed constructs explained consumers’ intention to adopt personalised nutrition, the overall performance of the theoretical model was good. Compared to the latent constructs, the effect of the service attributes on *Adoption Intention *was, however, small. The difference in the extent to which the latent constructs and service attributes were able to explain *Adoption Intention* may be caused by the design of this study and participants’ lack of knowledge about or relevance of personalised nutrition service attributes. Evaluating two of the 144 personalised nutrition services without being familiar with the full range of possible service attributes may have caused the within-participant measured effects of the latent constructs to dominate over the between-participant measured effects of the services attributes.

In relation to overall health, the present study examined consumers’ intention to adopt personalised nutrition services based on the perceived benefits of personalised nutrition advice compared to general nutrition advice. It is important to recognise that the benefits of improved overall health, in most instances, will only materialise if consumers adhere to the provided nutrition advice. Future research is needed to better understand the drivers and barriers of adherence to personalised nutrition advice. Important in this respect is also that some health benefits may be experienced shortly after engaging with a personalised nutrition service (e.g. increase in physical fitness), while other health benefits only materialise over a longer period of time (e.g. prevention of chronic diseases). Lack of direct feedback on long-term health improvement may, however, reduce motivation to adhere to the advice. Future research should identify whether and how direct feedback may contribute to advice adherence, either through directly perceivable improvements related to, for instance, physical performance, and/or the use of more dynamic assessments enabled by wearable devices capable of monitoring relevant biomarkers.

## Conclusion

This study confirmed a robust and Europe-wide applicable cognitive model showing how the *Privacy Calculus* and its antecedents determine consumers’ intention to adopt personalised nutrition services. For theory, the model implies that consumers’ intention to adopt personalised nutrition services depends more on perceptions of *Personalisation Benefit* than on perceptions of *Privacy Risk*. At the practical level, this implication suggests that to consolidate adoption, providers of services that require highly intrusive personal information such as DNA should pay attention to possible privacy risks that may keep consumers from engaging with their service. Service providers may reduce consumers’ *Privacy Risk *perceptions by, where possible, using less intrusive types of personal information such as lifestyle information and phenotypic information, or alternatively, and offer the option of using pseudonyms to anonymise data. Furthermore, it is important to more strongly emphasise and communicate the benefits of engaging with personalised rather than with non-personalised nutrition services, particularly how and why DNA profiling contributes to superior nutrition advice. Finally, to increase consumers’ perception of *Personalisation Benefit*, service providers should optimise the effectiveness of their service. Promising tools that may help increase service effectiveness are face-to-face communication and regular meetings.
